# Primary Biatrial Cardiac Rhabdomyosarcoma

**DOI:** 10.21470/1678-9741-2018-0414

**Published:** 2020

**Authors:** Tetsuro Uchida, Yoshinori Kuroda, Mitsuaki Sadahiro

**Affiliations:** 1 Second Department of Surgery, Faculty of Medicine, Yamagata University, Yamagata, Japan.

**Keywords:** Rhabdomyosarcoma, Heart Neoplasms, Sarcoma, Heart Atria, Prognosis, Female, Middle Aged

## Abstract

Primary malignant neoplasms of the heart are rare. Cardiac rhabdomyosarcoma is the second most common primary sarcoma. We report a rare case of a 49-year-old woman with a huge biatrial cardiac rhabdomyosarcoma treated by performing surgical resection followed by salvage chemotherapy for local recurrence. Cardiac sarcoma that occupy both atria are extremely rare. Although the prognosis of cardiac rhabdomyosarcoma is dismal, surgical resection should be recommended as a first line therapy to clarify the diagnosis and to relieve symptoms associated with the tumor.

**Table:** 

Abbreviations, acronyms & symbols	
MR	= Mitral regurgitation

## INTRODUCTION

Primary cardiac tumors are rare and have an incidence of 0.01-0.28%^[1]^. Approximately 1/4 of all primary cardiac tumors are malignant, with rhabdomyosarcoma being the second most common primary cardiac sarcoma^[1]^. Cardiac rhabdomyosarcoma is an aggressive tumor with high mortality rates. Cardiac sarcoma that occupy both atria are extremely rare^[2]^. Herein, we describe a rare case of huge biatrial cardiac rhabdomyosarcoma treated by surgical resection followed by salvage chemotherapy for local recurrence.

## CASE REPORT

**Fig. 1:**
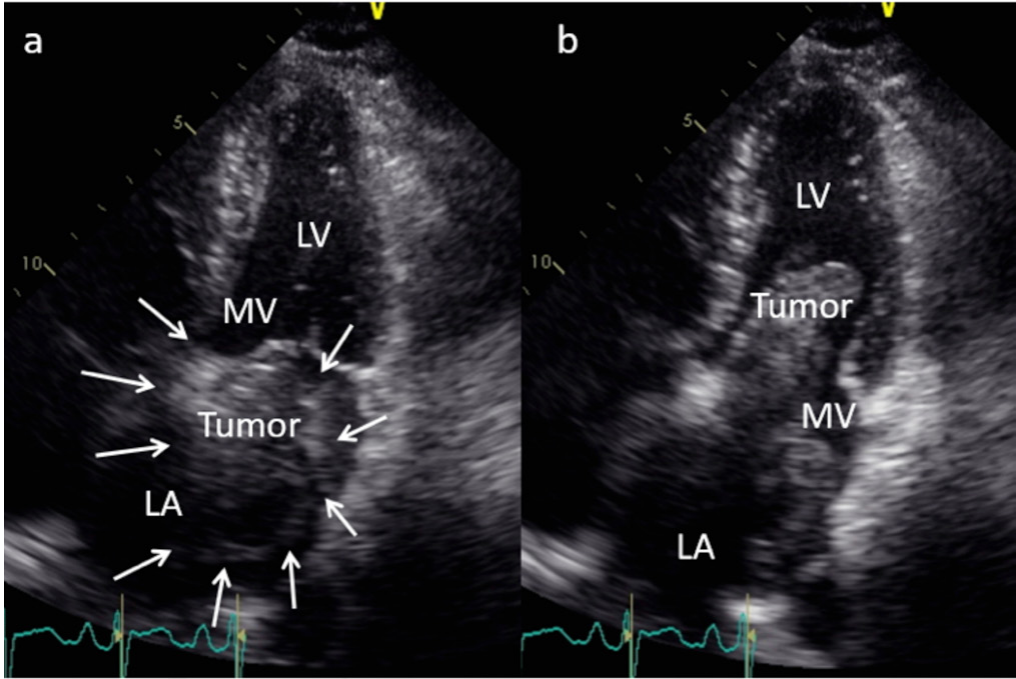
A) Echocardiography showed a well demarcated huge mass in the left atrium (arrows); B) Left atrial tumor herniated to the mitral valve orifice; LV=left ventricle; MV=mitral valve; LA=left atrium

On admission, she presented with dyspnea and an apical systolic murmur. Echocardiography revealed a huge mass in the left atrium (100 x 100 mm) that occupied most of the left atrial chamber (Figure 1A) and a right atrial tumor (20 x 10 mm) that was attached to the interatrial septum. Moderate grade mitral regurgitation (MR) was also observed (Figure 1B). Computed tomography showed a huge lobulated hypodense lesion in the left atrium. Surgical resection of tumors was indicated as a first line palliative therapy for space-occupying cardiac tumors.

**Fig. 2:**
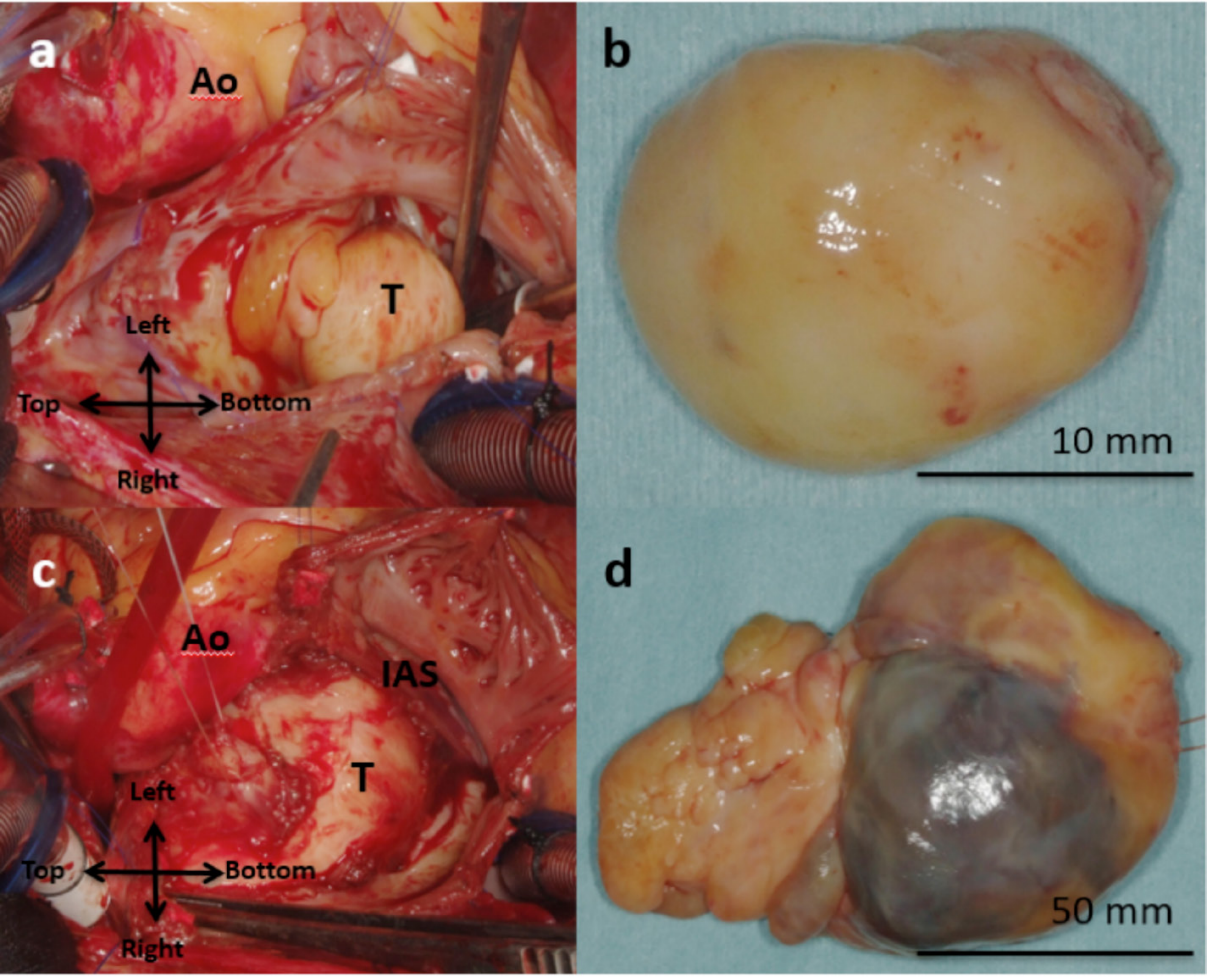
A) Right atrial tumor originated from the interatrial septum; B) The right atrial tumor was hard, smooth surfaced and white, measuring 20 x 10 mm; C) Left atrial tumor occupying the entire left atrial cavity. D) The left atrial tumor consisted of two globular encapsulated components measuring 105 x 63 mm and 41 x 30 mm respectively. Ao=ascending aorta; T=tumor; IAS=interatrial septum

The patient underwent surgery through a median sternotomy. Under cardiopulmonary bypass following aortic cross clamping, the right atrium was opened and a hard, smooth surfaced, white tumor originating from the ovarian fossa of the interatrial septum was removed with an origin of its stalk (Figures 2A and B). Via a wide opening of the interatrial septum and the roof of the left atrium, the narrowed cavity of the left atrium was entered. A huge solid tumor consisting of two globular yellow-grey encapsulated components, measuring 105 x 63 mm and 41 x 30 mm respectively, occupied the left atrial cavity (Figures 2C and D). The tumor stemmed from the posteromedial part of the mitral valve annulus and was removed with its stalk. The valve itself was normal and preoperative MR was considered to be due to the compression to mitral leaflets by the huge tumor. Intraoperative frozen section analysis suggested a diagnosis of malignant neoplasm. Defects of interatrial septum and left atrial wall were augmented and reconstructed using glutaraldehyde-treated autologous pericardium. The postoperative course was uneventful, and echocardiography showed trivial MR. Histopathological examination indicated that both atrial tumors were rhabdomyosarcoma and tumor margins were free of tumor.

Eight months later, intracardiac recurrence was detected. Echocardiography showed rapid left atrial tumor growth and gradual worsening of MR due to the obstructive effects of the enlarged tumor. Instead of radical resection of recurrent tumor, she received molecular-targeted therapy with pazopanib as a salvage chemotherapy. Although tumor progression was suppressed transiently during chemotherapy, it was abandoned due to considerable side effects. After recurrence, tumor growth was extremely rapid, and she died 13 months after surgical resection.

## DISCUSSION

Malignant neoplasms constitute ~25% of all primary cardiac tumors. Rhabdomyosarcoma is a relatively rare cardiac tumor. But it is the second most common malignant primary tumor of the heart following angiosarcoma^[1]^. In adults, only 65 cases of rhabdomyosarcoma have been reported; cardiac sites involved include the left atrium (55%), left ventricle (15.7%), right ventricle (15.7%), and right atrium (13%)^[3]^. Biatrial rhabdomyosarcoma, as in our case, is extremely rare^[2]^.

Differential diagnosis between benign cardiac tumor and sarcoma is important for determining appropriate treatment. It is difficult due to a lack of specific clinical symptoms and typical finding of preoperative examinations. In our case, valvular disease was suspected as a cause of progressive congestive heart failure. Following examination, the patient was diagnosed with biatrial tumors mimicking mitral valvular disease.

Although the prognosis after surgery is usually excellent in patients with benign cardiac tumors, survival from diagnosis is <1 year in most cardiac rhabdomyosarcoma cases, after radical surgical resection^[4]^. There is no consensus about how to treat cardiac rhabdomyosarcoma, because of small patient population and the benefits of surgery and postoperative chemotherapy or radiotherapy are still unclear, and long-term survival is poor even after multidisciplinary treatment^[5]^. Multi-treatment approach has no curative but palliative effect only, however, it is considered to contribute to patient’s prognosis. Although the prognosis of cardiac rhabdomyosarcoma is dismal, surgical resection should be recommended as a cornerstone of these therapeutic modalities to clarify the diagnosis, relieve symptoms, and improve short-term survival^[5]^.

**Table:** 

Author's roles & responsibilities	
TU	Substantial contributions to the conception or design of the work; or the acquisition, analysis, or interpretation of data for the work; final approval of the version to be published
YK	Final approval of the version to be published
MS	Substantial contributions to the conception or design of the work; or the acquisition, analysis, or interpretation of data for the work; final approval of the version to be published
